# University of Michigan - College of Dental Surgery

**Published:** 1882-05

**Authors:** 


### UNIVERSITY OF MICHIGAN—COLLEGE OF DENTAL SURGERY.

Seventh annual commencement.

The number of matriculates for the term was twenty-six:

The degree of D.D.S. was conferred on the foliowing members of the class:

| NAME. | RESIDENCE. | NAME. | RESIDENCE. |
|---|---|---|---|
| George P. Ashton | Warrensburgh, Mo. | Julien W. Lyon | Akron, O. |
| Wellington B. Banks | Detroit. | Hattie L. Martindale | Grand Rapids. |
| Frederick J. Barnes | Delaware, O. | Kate Cameron Moody | Mendota, Ill. |
| Fred. Thompson Bell | Aurora, Ill. | Frank S. Morrison | Martin's Ferry, O. |
| James C. Bush | Hardinsburgh, Ky. | Edward M. Nutting | Randolph, N. Y. |
| Wesley J, Campbell | Malone, N. Y. | Romeyn M. Paine | Springport. |
| Jennie M. Clark | Milwaukee, Wis | Charles F. Porter | Bay City. |
| Charles E. Cleveland | Chicago, Ill. | William H. Priestman | Neponset, Ill. |
| Edward C. Condict | Dover, N. J. | Joseph Lee Rose | Mobile, Ala. |
| Herbert Lee Davis | Fostoria, O. | Walter I. Southerton | Bay City. |
| Bernard John DeVries | Holland. | Edmund P. Stiles | Austin, Tex. |
| Charles Albert Eckert | Trenton, O. | Wilber A. Studley | Springville, N. Y. |
| Margaret Humphreys | Xenia, O. | Robley O. Sturgeon | Salem, O. |
| Henry A. Knight | Minneapolis, Minn. | Harry B. Tileston | Evansville, Ind. |
| Joseph B. Little | Fond du Lac, Wis. | Wm. A. B. Treadway | Norwich, Conn. |
| Harry M. Loughridge | Mansfield, O. | James M. Welch | Baltimore, Md. |

